# A Fast, Naked-Eye Assay for Varietal Traceability in the Durum Wheat Production Chain

**DOI:** 10.3390/foods9111691

**Published:** 2020-11-19

**Authors:** Giulia Cibecchini, Paola Cecere, Giorgio Tumino, Caterina Morcia, Roberta Ghizzoni, Paola Carnevali, Valeria Terzi, Pier Paolo Pompa

**Affiliations:** 1 Istituto Italiano di Tecnologia, Nanobiointeractions & Nanodiagnostics, Via Morego 30, 16163 Genova, Italy; giulia.cibecchini@iit.it (G.C.); paola.cecere@iit.it (P.C.); 2 Department of Chemistry and Industrial Chemistry, University of Genova, Via Dodecaneso 31, 16146 Genova, Italy; 3 Council for Agricultural Research and Economics, Research Centre for Genomics and Bioinformatics, Via San Protaso 302, 29017 Fiorenzuola d’Arda PC, Italy; giorgiotumino@hotmail.it (G.T.); caterina.morcia@crea.gov.it (C.M.); roberta.ghizzoni@crea.gov.it (R.G.); 4 Barilla S.p.A., Via Mantova 166, 43122 Parma PR, Italy; paola.carnevali@barilla.com

**Keywords:** durum wheat variety, genetic traceability, single nucleotide polymorphisms, colorimetric tests, authenticity, point of care test

## Abstract

The development of a colorimetric mono-varietal discriminating assay, aimed at improving traceability and quality control checks of durum wheat products, is described. A single nucleotide polymorphism (SNP) was identified as a reliable marker for wheat varietal discrimination, and a rapid test for easy and clear identification of specific wheat varieties was developed. Notably, an approach based on the loop-mediated isothermal amplification reaction (LAMP) as an SNP discrimination tool, in combination with naked-eye visualization of the results, was designed and optimized. Our assay was proven to be effective in the detection of adulterated food products, including both substitution and mixing with different crop varieties.

## 1. Introduction

Food traceability currently has a key role in the agrifood sector, bringing many benefits to both industries and consumers, such as supply chain optimization, improvement in food safety and quality, and an increase in controls and in the consumers’ confidence. Mandatory traceability regulations have been established by several countries with different level of stringency for both domestic and imported commodities and agrifood products (for a review, see Charlebois et al., 2014 [1]). Mandatory traceability actions are flanked by voluntary ones, characterized by the identification of standards related, for example, to the valorization of traditional food products and local economy development [2]. In this framework, the world leader in Italian pasta manufacturing has started a pilot study equipping its products with a digital passport, i.e., a QR code capturing the entire food journey from “field to fork” [3]. Such an approach can enhance transparency and safety, and creates a connection between the consumers and the regions where their food was produced. A pillar of Italian durum wheat pasta production chain is, however, the grain identity, which is mandatory at the species level. The use of durum wheat semolina is, in fact, compulsory for Italian pasta, in which the *Triticum aestivum* species is considered a contamination that must not exceed the 3% maximum level [4]. However, the quality of the end products can be related not only to species, but also to the cultivar [5]. At the industrial level, pasta is commonly made by mixtures of different durum wheat cultivars. At the same time, there is an increasing presence in the market of high-value mono-varietal pasta from both local producers and industrial ones. In such productions, there is great interest in developing a voluntary traceability system to identify specific varieties along the production chain.

Varietal fingerprinting approaches have been developed from mainly to protect intellectual property (IP) of wheat variety to guarantee plant breeders’ rights (PBR). Distinctness, uniformity, and stability are among the basic requirements for a variety. The traditional morpho-physiological descriptors, collected during the whole plant life cycle, are classically used for such evaluation purposes. In a further step, morphological descriptors have been flanked by biochemical markers, e.g., the isoforms of seed storage proteins. Such markers can be informative when applied to the grains, but are not effective on transformed products, in which technological processes can impair the proteins’ integrity. The subsequent advancement in varietal fingerprinting was based on the introduction of DNA markers that are independent of the environmental influence and available in theoretically unlimited numbers. Many classes of DNA molecular markers have been developed, ranging from inter simple sequence repeats (ISSRs) to amplified fragment length polymorphism markers (AFLP) and random amplified polymorphic DNA (RAPD), as reviewed by Pasqualone, 2011 [6]. SSRs (simple sequence repeats), considered the second generation of molecular markers and characterized by a simple technique, high polymorphism rate, and robustness, have been widely employed in wheat [7,8]. SNPs (single-nucleotide polymorphisms) are the third generation of molecular markers, with several advantages, including high frequency across the whole genome, ease of detection, and cost efficiency [9]. Classical techniques for SNP genotyping are currently sequencing, SNP array, or PCR-based methods, such as the restriction fragment length polymorphism (RFLF) and amplification refractory mutation system (ARMS). In particular, for the wheat genome, SNP arrays are available and permit the identification of 9000 to 90,000 polymorphisms [10,11,12]. Moreover, with the development of next-generation sequencing (NGS) technology, increasing numbers of SNPs have been discovered, building the basis for the development of diagnostic SNP barcodes [13]. Now, the “universalization and minimization of SNP number without compromising identification accuracy is the major challenge in development of varietal profile by rapid genotype assay”, as reported by Singh et al., 2019 [14]. This step of identifying a small panel of informative SNPs is accompanied by the need to have efficient and low-cost techniques for their identification in large numbers of samples. However, classical methods are time consuming, expensive, and require costly instrumentations and specialized personnel, limiting their practical applicability for quality control screenings in the food supply chain. A rapid, on-site technique for SNP discrimination would thus be of great industrial interest. In this work, starting from a detailed SNP analysis on a panel of durum wheat, we set up a pilot study aimed at developing a point of care (POC) testing method to track, in a fast and inexpensive way, the presence of a specific wheat variety (Aureo) in grains and flours. In particular, we first identified an SNP allele specific for the focus variety, then we developed a portable colorimetric method based on the loop isothermal amplification reaction (LAMP) technique [15,16]. Our assay enables on-site, rapid quality control analyses by untrained personnel through simple visual inspection.

## 2. Materials and Methods

### 2.1. Plant Material and DNA Extraction

The following 28 durum wheat varieties were used: Iride, Rusticano, Saragolla, Odisseo, Maestrale, Bronte, Antalis, Fabulis, Core, Svevo, Orizzonte, Aureo, Achille, Monastir, Claudio, Tirex, Pigreco, Normanno, Marco Aurelio, Relief, Miradoux, Babylone, Simeto, Anvergur, Navigator, Levante, Kyle, and Kronos. Moreover, the tetraploid *Triticum turanicum*, variety QK-77 (traded as Kamut^®^), was included in the analysis. All of the varieties were certified foundation seeds produced directly from the breeder responsible for their maintenance in purity. Ten certified seeds of each variety were sown in duplicates in pots, and the foliar tissues were harvested at the three-leaves stage. DNA samples were isolated and purified using the cetyl trimethyl ammonium bromide method [17]. The quality check, quantification, and concentration adjustment were accomplished with a NanoDrop2000C Spectrophotometer (Thermo Fisher Scientific, Monza, Italy). The concentration of each sample was adjusted to 50 ng/μL. Two biological replicates of DNA extracts were used for each variety.

### 2.2. Genotyping by Sequencing and SNP Data Analysis

Purified DNA samples (1 μg for each sample) were sent to Diversity Arrays Technology Pty Ltd. (http://www.diversityarrays.com/, Canberra, Australia) for sequencing, and SNP marker identification was done by DArTseq genotyping. Sequences of the genomic representations were obtained on a HiSeq2500 instrument.

The set of SNP markers generated by DArTseq was curated, removing markers with more than 25% of missing values (null alleles were considered as missing). SNPs with low frequent alleles were kept in the dataset to allow the research of private alleles of Aureo. This resulted in a dataset containing 20,198 SNPs and 58 samples (two genotyping replicates for each variety). After marker curation, all samples had a percentage of missing values below 10% and a percentage of heterozygous calls below 12%. Genetic distances were calculated based on simple matching.

A custom R script was used to select SNPs with no missing values and a private allele of both replicates of Aureo. Candidate SNPs were mapped on the durum wheat genome sequence [18] by BLAST (Basic Local Alignment Search Tool). Starting from a panel of 11 candidates, an SNP mapped on chromosome 7A was selected for further analyses.

### 2.3. Primer Design

LAMP primers were designed from the 7A chromosome of durum wheat. Primer sequences are shown in Table 1:

### 2.4. LAMP Reactions for RealTime

LAMP reactions were performed in 25 μL of a mixture containing 1.6 μM each of inner primers, 0.4 μM each of outer primers, 0.8 μM of loop primers (Integrated DNA Technologies, Coralville, IA, USA), 1 M of betaine (VWR International SRL, Milano, Italy), 2.5 μL of 10× LAMP buffer (200 mM of Tris-HCl, 100 mM of (NH_4_)2SO_4_, 20 mM of MgSO_4_, 500 mM of KCl, and 1% *v*/*v* Tween 20), 2 mM of MgSO_4_, 0.8 mM each of dNTPs (Promega, Madison, WI, USA), 0.112 U/μL of Bst 2.0 WarmStart DNA Polymerase (New England BioLabs, Ipswich, MA, USA), and 5 μL of DNA template at the concentration of 5 ng. DNA-free LAMP reactions were included as negative controls. Amplification reactions were performed using forward and backward inner primers (FIP and BIP), forward and backward outer primers (F3 and B3), and one loop primer (LoopT or LoopG). For real-time fluorescent LAMP, 1/50,000 diluted SYBR Green (Thermo Fisher Scientific, Waltham, MA, USA) was pre-added to the reaction mix. Real-time amplifications were performed on an Applied Biosystem real-time instrument (Thermo Fisher Scientific, Waltham, MA, USA) with StepOne Software v2.3 at 63 °C.

### 2.5. LAMP Reactions for Colorimetric Assay

LAMP colorimetric reactions were performed as reported above, using 1 M of betaine (VWR International SRL, Milano, Italy), 2.5 μL of 10× LAMP buffer (100 mM of (NH_4_)2SO_4_, 20 mM of MgSO_4_, 500 mM of KCl, and 1% *v*/*v* Tween 20), 2 mM of MgSO_4_, 0.8 mM each of dNTPs (Promega, Madison, WI, USA), 0.112 U/μL of Bst 2.0 WarmStart DNA Polymerase (New England BioLabs, Ipswich, MA, USA), and 5 μL of DNA template at the concentration of 5 ng. DNA-free LAMP reactions were included as negative controls. For visualized detection, 0.06 mM of Cresol Red (Sigma-Aldrich, St. Louis, MO, USA) was pre-added to the reaction mix. The amplification efficiency was verified by color changing of the reaction mix. Colorimetric LAMP reactions were performed on a T100 Thermal Cycler (BIO-RAD, Hercules, CA, USA) at 63 °C for 1 h, followed by heat inactivation at 90 °C for 2 min.

## 3. Results and Discussion

In this work, we aimed at developing a rapid assay for Aureo varietal discrimination based on a single SNP suitable for POC settings. A preliminary genotyping study had the objective of identifying a set of SNPs capable of discriminating Aureo within a panel of 29 wheat varieties. The DArTseq methodology applied on this panel of varieties produced a set of 20,198 good-quality SNPs (with a percentage of missing values below 25%). In order to develop a single-SNP assay, we selected SNPs tagging a private allele of Aureo. The genotyping of two replicates per variety reduced the risk of selecting a private SNP allele caused by a genotyping error. The average genetic distance between two replicates was 98.5% (simple matching), suggesting a genotyping error rate of 1.5% on average. A set of 11 candidate SNPs carrying a private allele of Aureo was identified, with six of them presenting no missing values. To ensure the selection of a private SNP allele, the SNP marker to be used for development of the LAMP assay was selected among those six SNPs with complete genotypes. The assay developed in this study was based on an SNP located on Chr 7A.

The low missing rate and the number of SNPs generated in this study enabled the identification of six SNPs as potential candidates for the assay. This was sufficient for developing our single-SNP approach, which aimed at the discrimination of a variety within a relatively small panel. Such an approach would be very suitable for specific industry requirements.

Such a need is relevant in several high-value agrifood products. In Italy, for example, some PGI (protected geographical indication) products, such as Amarene brusche di Modena, Ciliegia di Vignola, and Pera dell’Emilia-Romagna, are regulated by product specifications that require the use of specific varieties. Moreover, worldwide wine production is linked to the use of specific grape varieties. Various wine laws include appellation-based regulations that cover not only the area of production and the wine-making practices, but even the permitted grape varieties. European PDO (protected designation of origin) and PGI are examples of such regulations directed to guarantee that a certain wine is made from a percentage of grapes belonging to a specific variety or to a specific panel of varieties. However, the genotyping effort required to identify a private SNP allele might be higher in situations with a larger panel of varieties or lower genetic diversity. To overcome this issue, a multi-SNP assay could be developed, since a private allele can be tagged more easily using more than one SNP.

In this framework, LAMP has been recently proven to be an interesting tool to achieve SNP genotyping [19]. In general, LAMP strategies for SNP detection are based on suitable modifications of the primer design, in which two versions of the same primer are typically used to distinguish both the wild-type and the polymorphic allele. The most common approaches exploit the design of the inner primers, hybridizing to the polymorphic or the wild-type base with the 5′ end of both FIP and BIP [20,21], or with the second base at the 5′ end of FIP and BIP [22]. Other methods use allele-discriminating outer primers, placing the mutation at the 3′ end of the F3 primer [23] or one inner primer with the mutated base at the 5′ end [24], or exploit peptide nucleic acid-locked nucleic acid (PNA-LNA) mediated LAMP reactions [25]. In several cases, however, these approaches lead to high background and low discrimination efficiency due to the poor inhibition of the non-specific amplification. Herein, we focused on the optimization of a recently published methodology proposed for the detection of salivary SNPs [26] based on the use of loop primers as allele-discriminating primers. In this strategy, two reactions are performed in parallel, one with the loop primer hybridizing with the wild-type base and one with the loop primer perfectly matched to the polymorphic one. Since loop primers are designed short, one mismatch significantly destabilizes the annealing, and the obtained effect is comparable to that of a reaction performed without loop primers. For instance, in the case of a wild-type target gene, the reaction performed with the polymorphic loop primer resulted in a strongly decreased amplification rate, creating a significant time gap between the two reactions [26].

In this work, we optimized this approach for Aureo varietal discrimination, exploiting the high tolerance of LAMP to contaminants and the possibility of instrument-free visualization of the results through pH-sensitive dyes [27,28], which together may provide an excellent portable tool for colorimetric SNP discrimination in food products/ingredients in industrial and supply chain settings.

**Figure 1:**
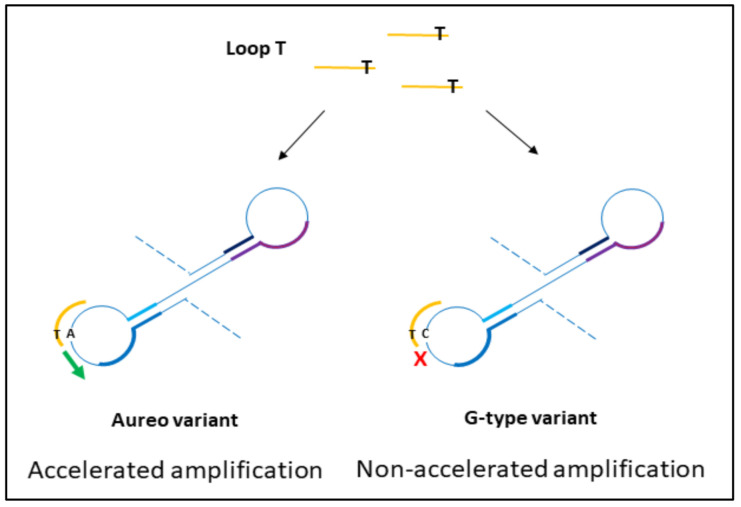
Schematic representation of the hybridization of loop primers to amplicon loops from durum wheat variants. During the exponential phase of loop-mediated isothermal amplification (LAMP) reaction, the perfect match between loop T and the complementary region on the Aureo intermediate loops accelerates the amplification, while the presence of the single mismatch between loop T and G-variant amplicon loops causes the short primer destabilization and a significant reaction delay. The results are reversed for loop G mediated reactions.

**Figure 2:**
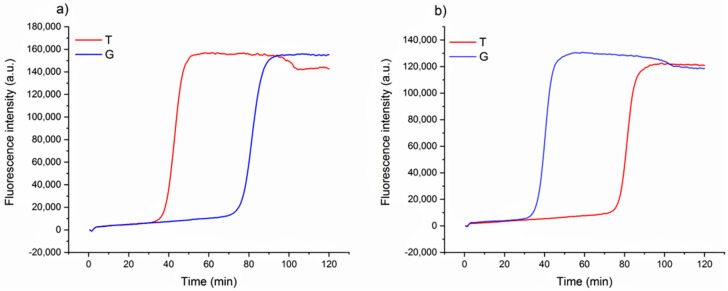
Real-time fluorescence detection of genetic variants of wheat. LAMP reaction with loop primer T (red line) and G (blue line) of Aureo variant genome (**a**) and one G-type variant genome (Marco Aurelio) (**b**). The time gap between fast and slow reactions is ca. 40 min. All of the reactions were performed at 63 °C.

The design of loop primers has a pivotal role in this strategy. In particular, the approach relies on the stabilization of the annealing between short loop primers and the perfectly complementary sequence, while the non-specific one is disfavored. Loop primers act as accelerating primers, speeding up the onset of the amplification. Hence, the reaction in which the loop primer is perfectly hybridized will be properly enhanced, unlike the one with the unstable annealing of the primer, which will be delayed (Figure 1). A single mismatch located on the short primer will significantly impair the annealing, reducing the efficiency of the amplification. Loop primers were constructed as the following: loop primer T, complementary to the SNP that identifies Aureo variant, and loop primer G, perfectly matching the SNP distinctive of all of the other durum wheat variants (see also Experimental). For each sample, two reactions were carried out in parallel to ensure that the same target was tested using both loop primer sets T and G. The results of the SNP-sensitive LAMP reactions on real wheat samples, followed in real-time, are reported in Figure 2. The reaction with the Aureo variant had a perfect annealing when performed with loop primer T, so in this case, the amplification was properly enhanced (ca. 40 min, see Figure 2a). On the other hand, with the same template, the reaction performed with loop primer G was destabilized and significantly delayed. The resulting time gap between the two reactions was about 40 min, allowing for an excellent discrimination. At the same time, the reaction performed on a non-Aureo genetic variant (Marco Aurelio, Figure 2b) revealed the inverted behavior, with similar high temporal discrimination between the fast, matched (G) and non-matched (T) reactions. This preliminary result demonstrated that, in this configuration, a single mismatch can properly modulate the amplification efficiency of LAMP; consequently, it is possible to identify one specific variant of durum wheat with high discrimination effectiveness.

**Figure 3:**
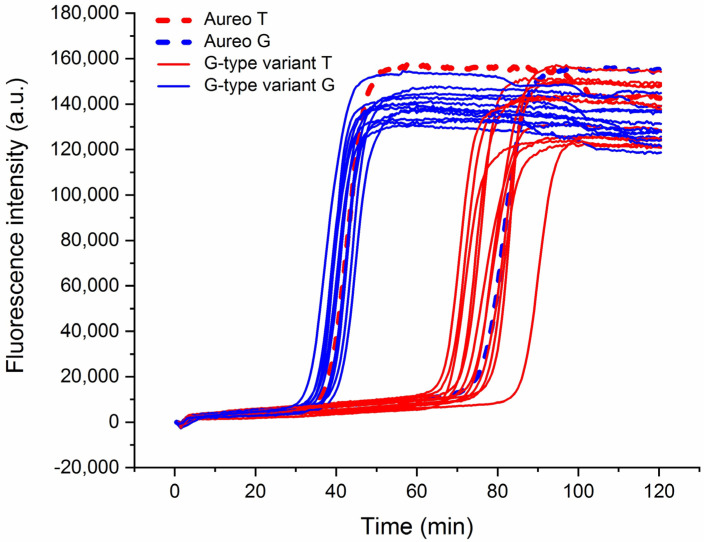
Real-time fluorescence detection of 13 genetic variants of wheat. LAMP reactions with loop primer T (red lines) and loop primer G (blue) of Aureo and G-type variants (Aureo: dashed lines). The time gap between all of the fast and all of the slow reactions was 35–40 min. All of the reactions were performed at 63 °C.

This strategy was applied to the assessment of a wide range of different wheat varieties to analyze the reliability of the technique in a real scenario of quality control. Interestingly, the method was able to efficiently discriminate all of the 13 tested varieties of wheat versus Aureo (Figure 3), with an average time gap of ca. 35–40 min between the fast and the slow reactions. Moreover, it is notable that the time gap was nearly constant among all the tested cases.

**Figure 4:**
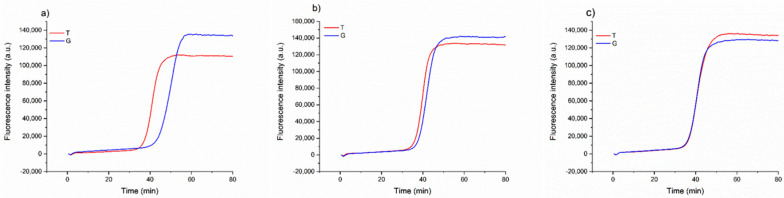
Real-time fluorescence detection of a simulated adulteration, in which a sample of Aureo was mixed with different proportions of a G-type variant (Babylone). (**a**) 90% of Aureo with 10% of G-type variant; (**b**) 75% of Aureo with 25% of G-type variant; (**c**) 50% of Aureo with 50% of G-type variant. The LAMP reactions were performed with loop primer T (red line) and G (blue line) at 63 °C.

Once we assessed that the method could consistently detect the possible substitution of the Aureo with another variety, we investigated a more complex scenario of food fraud, in which the Aureo species is adulterated (mixed) with another variety, with different levels of contaminations. This is important in industrial settings to guarantee the mono-varietal nature of some productions. To this purpose, different mixtures of Aureo with decreasing contaminations of a G-type variant (from 50 to 10%) were prepared to probe the sensitivity of the strategy to also detect minor adulterations. As reported in Figure 4, the method can effectively discriminate the 50/50 (Aureo/G-type) mixture, with the two reactions displaying similar efficiency. In such a case, the two reactions both behave like fast reactions, because the half amount of template of each variant is sufficient for the optimized functioning of the amplification (Figure 4c). Overall, the mixture appeared as a heterozygous sample. Comparable results were obtained with a 25% contamination with the G-type variant (Figure 4b). Although the amount of G-type genome was lower, the reaction with loop primer G worked efficiently, exhibiting a negligible delay. Interestingly, our LAMP test was also able to discriminate an adulteration with the G-type variant as low as 10% (Figure 4a). In this latter case, we observed a small delay of the loop primer G reaction due to the significantly lower (10%) amount of G-type variant present in the mixture. However, the test behaved very differently with respect to the result obtained with 100% pure Aureo mixture, overall indicating that the proposed strategy enables accurate discrimination of relevant adulterations, including both substitution and mixing of wheat varieties (see also in the following).

**Figure 5:**
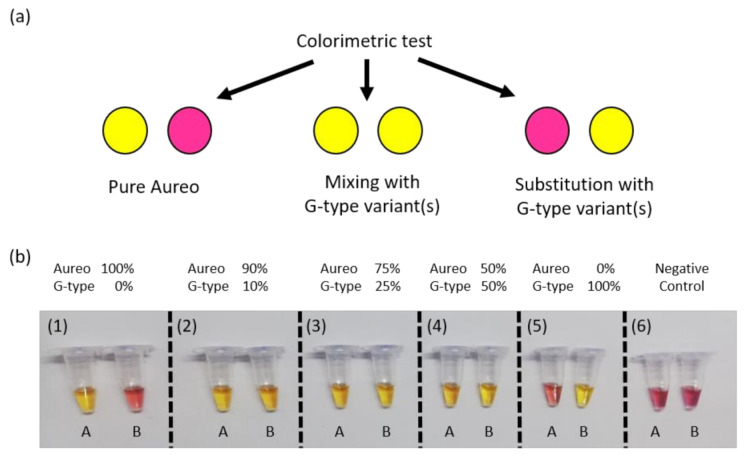
(**a**) Schematic of the colorimetric test displaying the possible yellow/purple color combinations: pure Aureo variety (yellow/purple), adulterated mixture with G-type varieties (yellow/yellow), or fully substituted product (purple/yellow). (**b**) Colorimetric detection of possible adulteration cases of Aureo with a G-type wheat variant (here, Babylon is used as an example). LAMP reactions with loop primer T (tube A) and loop primer G (tube B) were all performed at 63 °C for 1 h. From left to right, targets were composed of 100% of the Aureo variant of wheat (sample 1), then mixtures of Aureo and G-type variant (from 10 to 100%, samples 2–5), and a negative control (sample 6).

After characterizing and optimizing the performance of our SNP test, we attempted to develop a simplified, instrument-free colorimetric assay with a naked-eye readout. Visual interpretation of the varietal discrimination was made possible by the reproducible and large time gap achieved between the fast and the slow reactions, in combination with pH-sensitive dyes that could provide a clear color change of the reaction tubes upon target amplification [20,21]. In particular, we used Cresol Red dye, which shifts from purple to yellow during amplification, and a reaction time of 1 h to fully discriminate between the two reactions. Based on the results showed above (Figure 2, Figure 3 and Figure 4), the fast reaction should display clear color change after 1 h, unlike the unmatched slow reaction. This was expected to enable visual discrimination of all of the adulteration cases analyzed so far. In line with previous experiments, reactions were carried out in tube A with loop primer T and in tube B with loop primer G. In more detail, as reported in the scheme in Figure 5a, the combination of the yellow/purple colors of the two reactions performed in parallel allowed detecting the possible scenarios, i.e., pure Aureo, the presence of contaminations (variety mixing), or full substitution. We tested all of the contamination cases, mixing Aureo with different fractions of a G-type variety (from 0 to 100%). What’s noteworthy is that the colorimetric visual test was proven to be effective and highly specific (Figure 5b). In particular, in the case of the pure Aureo variant, the reaction performed with loop primer T turned yellow (positive result), while the reaction performed with loop primer G remained purple (negative result) (Figure 5b, sample 1). On the contrary, the full substitution of Aureo with a G-type variant exhibited the inverted result, with tube A appearing purple and tube B yellow (Figure 5b, sample 5). Interestingly, all of the adulterated samples with different levels of contaminations (down to 10% adulteration) were clearly detected, with both of the tubes exhibiting yellow color (samples 2–4) due to the amplification of both genomic variants. Hence, this direct and instrument-free strategy was proven to be specific and had enough sensitivity for quality control purposes, with the visual inspection of the results being an additional advantage for on-field applications.

**Figure 6:**
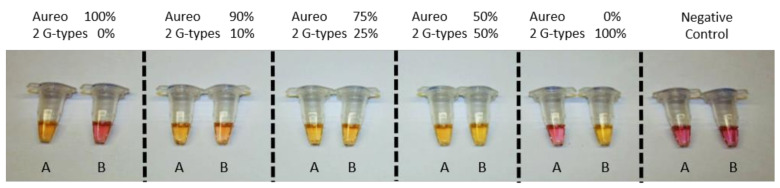
Colorimetric detection of a complex adulteration of Aureo with two G-type wheat variants. LAMP reactions with loop primer T (tube A) and loop primer G (tube B) were all performed at 63 °C for 1 h. From left to right, targets were composed of 100% Aureo variant, then mixtures of Aureo and two G-type variants (Babylon + Marco Aurelio) with a total contamination from 10 to 100%, and a negative control.

To completely validate our system, a more complex case of adulteration was tested, in which the Aureo was mixed with two different G-type varieties of wheat (from 50 to 10% total contaminations). Remarkably, also in this case, the strategy revealed its high specificity, being able to correctly identify the different genomes in a complex mixture and even in the situation in which the total amount of the two contaminations was as low as 10% (Figure 6).

## 4. Conclusions

In conclusion, we designed a single-SNP discriminating strategy to correctly identify mono-varietal productions based on Aureo, and we set up a direct colorimetric test able to discriminate, by simple visual inspection, the purity or the adulteration of Aureo with a wide range of G-type variants. The test exhibited high efficiency and specificity in complex cases of adulteration, in which different varieties were mixed, even at a 10% level of contamination. The isothermal nature of the LAMP amplification together with the naked-eye readout of the results make this approach potentially very useful in real quality control applications in the industry and food supply chain.

## Figures and Tables

**Table 1:** Names and Primer sequences used in the study.

Name of Primers	Sequence (5′–3′)
FIP	TGCAGTGACTGATTGTACTGTCCACCACTTCCTCAGGTA
BIP	GTACTGCACTACTGCACCATTCTCGCCTGCAAACACAC
F3	CTGCCGTTGCCAACA
B3	TATCCGCACGCACC
LoopT	TGCAGGCGATG
LoopG	GCAGGCGAGG
